# Modulation of genomic and epigenetic end-points by celecoxib

**DOI:** 10.18632/oncotarget.26062

**Published:** 2018-09-14

**Authors:** Alberto Izzotti, Sebastiano La Maestra, Rosanna T. Micale, Alessandra Pulliero, Marta Geretto, Roumen Balansky, Silvio De Flora

**Affiliations:** ^1^ Department of Health Sciences, University of Genoa, 16132 Genoa, Italy; ^2^ IRCCS Ospedale Policlinico San Martino, 16132 Genoa, Italy; ^3^ National Center of Oncology, 1756 Sofia, Bulgaria

**Keywords:** celecoxib, cigarette smoke, mouse models, DNA damage, microRNAs

## Abstract

Celecoxib, a nonsteroidal anti-inflammatory drug that selectively targets cyclooxygenase-2, is a promising cancer chemopreventive agent. However, safety concerns have been raised in clinical trials evaluating its ability to prevent colorectal adenomas. The rationale for the herein reported studies was to analyze genomic and epigenetic end-points aimed at investigating both the chemopreventive properties of celecoxib towards cigarette smoke-associated molecular alterations and its possible adverse effects. We carried out three consecutive studies in mice treated with either smoke and/or celecoxib. *Study 1* investigated early DNA alterations (DNA adducts, oxidative DNA damage, and systemic genotoxic damage) and epigenetic alterations (expression of 1,135 microRNAs) in lung and blood of Swiss H mice; *Study 2* evaluated the formation of DNA adducts in lung, liver, and heart; and *Study 3* evaluated the expression of microRNAs in 10 organs and 3 body fluids of ICR (CD-1) mice. Surprisingly, the oral administration of celecoxib to smoke-free mice resulted in the formation of DNA adducts in both lung and heart and in dysregulation of microRNAs in mouse organs and body fluids. On the other hand, celecoxib attenuated smoke-related DNA damage and dysregulation of microRNA expression. In conclusion, celecoxib showed pleiotropic properties and multiple mechanisms by counteracting the molecular damage produced by smoke in a variety of organs and body fluids. However, administration of celecoxib to non-smoking mice resulted in evident molecular alterations, also including DNA and RNA alterations in the heart, which may bear relevance in the pathogenesis of the cardiovascular adverse effects of this drug.

## INTRODUCTION

Nonsteroidal anti-inflammatory drugs (NSAIDs) are extensively used worldwide for the treatment of inflammation-related diseases. Among other applications, these agents provide a promising approach to the prevention of cancer, also including smoking-associated cancers [1]. The rationale for such a strategy is that chronic inflammation plays a key role at different stages of the carcinogenesis process [2, 3] and is crucial in tobacco smoke carcinogenesis [4]. NSAIDs interfere with the metabolism of arachidonic acid, a ω-6 essential fatty acid that is the substrate for different enzyme systems, including cyclooxygenases (COX), lipoxygenases, and cytochromes P450. Of the two COX enzymes, COX-1 is the housekeeping isoform, and the prostaglandins derived from COX-1 are involved in the homeostatic maintenance of the gastric mucosa. In contrast, COX-2 is the inducible isoform, expressed in response to certain stimuli such as mitogens, cytokines and growth factors, which has a pro-inflammatory function [5].

Based on these mechanistic premises, selective COX-2 inhibitors, called coxibs, have been developed to improve gastrointestinal (GI) safety and tolerability. They include drugs such as celecoxib, rofecoxib, valdecoxib, etoricoxib, and lumiracoxib. In particular, celecoxib (4-[5-(4-methylphenyl)-3-(trifluoromethyl)-1H-pyrazol-1-yl] benzene-1-sulfonamide, CAS 169590-42-5) was the first selective COX-2 inhibitor approved to treat patients with rheumatism and osteoarthritis. Upper GI complication rates in clinical trials have been reported to be significantly lower for celecoxib than for traditional nonselective NSAIDs [6–8]. While several coxibs have been withdrawn from the market due to safety concerns, and especially to cardiovascular adverse events [9, 10], celecoxib continues to be available for use in many countries [11]. This coxib is approved by the US Food and Drug Administration (FDA) ( https://www.fda.gov/ForConsumers/ConsumerUpdates/ucm240959.htm).

It has been shown that COX-2 downregulation inhibits colorectal carcinogenesis [10]. Moreover, based on reports that suggest an association between cigarette smoke (CS) and COX-2-associated risk to develop cancer, celecoxib is under scrutiny for its possible ability to prevent cancer and for antagonizing the carcinogenic properties of CS, the most important threat to human health. In humans, a case-control study showed that use of celecoxib and other coxibs reduces the lung cancer risk [12]. A trial of celecoxib in former smokers decreased the bronchial proliferation, evaluated by measuring the Ki-67 labeling index, and reduced lung nodules on computed tomography [13]. In mice, administration of celecoxib reduced oxidative stress-mediated alterations, due to its ability to boost the antioxidant defense system [14]. Coxibs and other NSAIDs are being tested in animal models of cancer chemoprevention [15]. Celecoxib reduced pulmonary inflammation but failed to affect lung tumorigenesis in mice treated with either 3-methylcholanthrene-butylated hydroxytoluene or urethane [16], while it prevented *N*-butyl-*N*-(4-hydroxybutyl) nitrosamine-induced bladder carcinomas in rats [17]. We showed that administration of celecoxib after weanling to Swiss H mice, which had been exposed to mainstream cigarette smoke (MCS) since birth and thereafter kept in filtered air for an additional 3.5 months, results in a variety of protective effects towards MCS-related carcinogenesis [18]. In particular, celecoxib significantly attenuated MCS-induced alterations of inflammatory nature, including pulmonary emphysema, alveolar epithelial hyperplasias and lung microadenomas as well as urinary tract hyperplastic lesions. Moreover, the drug attenuated the yield of lung adenomas and showed some involvement in lowering the progression to cancer in the lung. However, in the same study celecoxib showed some hepatotoxicity in MCS-exposed mice [18], which is consistent with the demonstration that mediators derived from COX-2 have an important hepatoprotective function and accordingly the risk of drug-induced liver injury may be increased by COX-2 inhibition [19].

The herein reported studies aimed at investigating both the chemopreventive properties of celecoxib in MCS-exposed mice and, at the same time, the possible adverse effects of this drug in smoke-free mice. To this purpose, we carried out three consecutive studies by using preclinical models that evaluated genomic and epigenetic end-points. In particular, the first study, referred to as *Study 1*, investigated early genomic alterations related to treatment of mice early in life with MCS and/or celecoxib. DNA alterations were evaluated in terms of bulky DNA adducts and oxidative DNA damage in lung and of systemic genotoxic damage, and epigenetic alterations were evaluated in terms of expression of microRNAs (miRNAs) in both lung and blood. This is the same murine model that we have extensively used to demonstrate, in the medium term, the carcinogenicity of MCS [20] and its modulation by a variety of putative cancer chemopreventive agents [21], also including celecoxib itself [18]. One of the findings of that study was that, while inhibiting the formation of DNA adducts in MCS-exposed mice, celecoxib induces the evident formation of DNA adducts in the lung of smoke-free mice. In order to validate the results of this study, we carried out a second study (*Study 2*) that specifically evaluated the selective formation of DNA adducts in the lung, liver, and heart of mice as related to exposure to MCS and administration of celecoxib. The third study (*Study 3*) was a quite extensive experiment that evaluated the expression of 1,135 miRNAs in 10 organs as related to the oral administration of celecoxib to adult mice, either smoke-free or MCS-exposed. Three body fluids were also examined, because extracellular miRNAs could serve as molecular biomarkers for diagnostic purposes applicable to both prevention and therapy of human diseases. The epigenetic alterations produced by MCS in this model, in the absence of chemopreventive agents, have previously been reported [22].

## RESULTS

### Study 1. Evaluation of genomic and epigenetic end-points in the lung and blood serum of newborn Swiss H mice as related to treatment with celecoxib and/or exposure to MCS

#### Survival and body weights

All mice survived after 10 weeks. The body weights in sham-exposed mice at weanling (about 4 weeks) were 23.9 ± 0.75 g in males and 18.7 ± 0.87 g in females and grew progressively until the 10th week of life up to 40.0 ± 0.74 g and 29.2 ± 0.56 g, respectively. From the 3rd week onwards exposure to MCS resulted in a slight but statistically significant loss of body weight, which after 10 weeks was 33.9 ± 0.62 g in males (*P <* 0.001 as compared to sham) and 27.3 ± 0.58 g in females (*P <* 0.05). As compared with sham-exposed mice, administration of celecoxib did not significantly affect the body weight, which was 37.2 ± 0.97 g in males and 27.8 ± 0.80 g in females. Celecoxib did not change the slight body weight loss caused by MCS, which was 34.6 ± 1.55 g in males and 26.7 ± 0.56 g in females. These figures were significantly lower than those recorded in sham-exposed mice (*P <* 0.05 in both genders).

### Bulky DNA adducts in the lung

**Figure 1:**
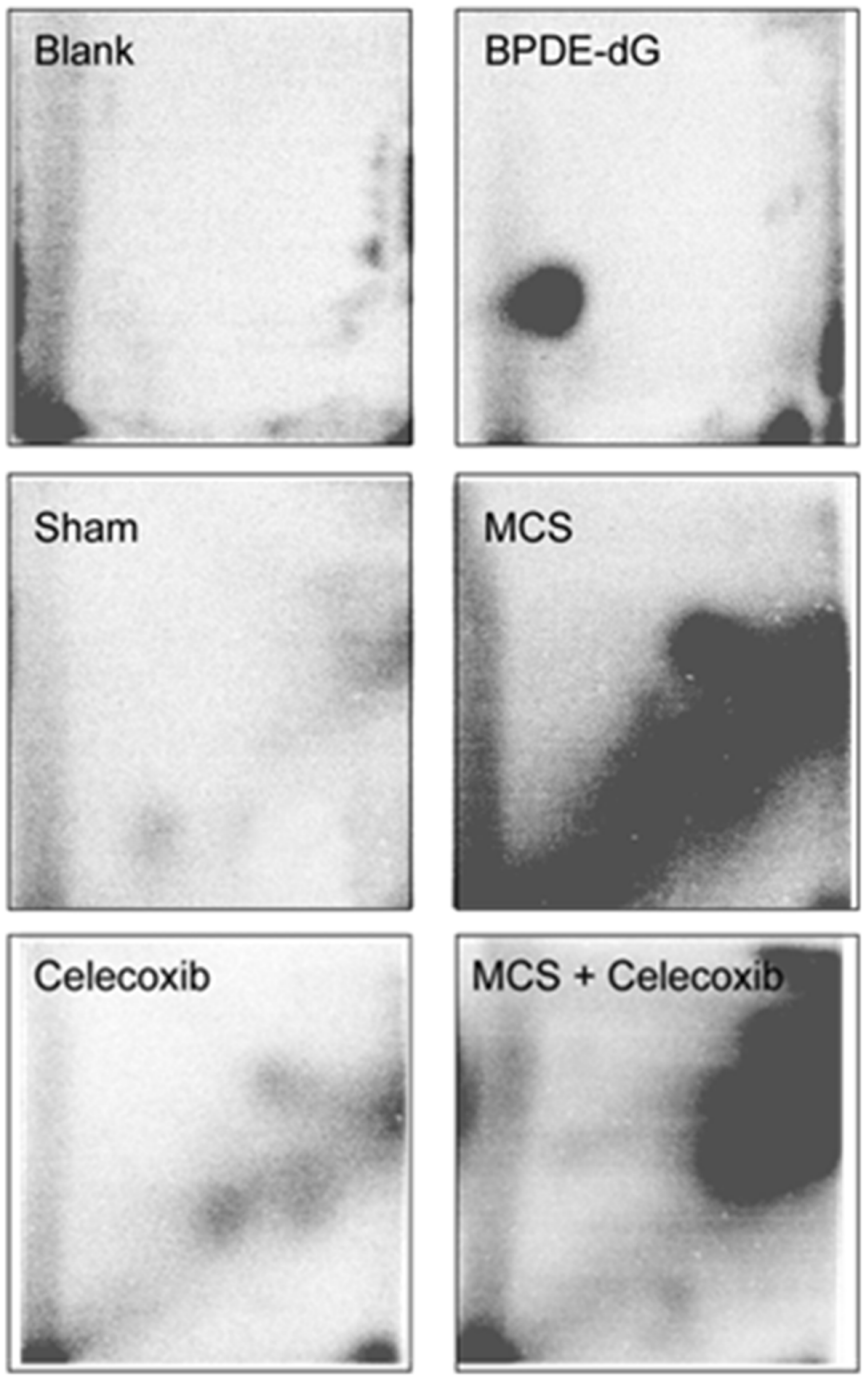
Examples of ^32^P autoradiographs obtained by testing a blank, a positive control (BPDE-dG), and the lung DNA from Swiss H mice, either untreated (sham-exposed) or treated with celecoxib for 6 weeks after weanling or exposed to MCS for 10 weeks since birth or exposed to MCS and treated with celecoxib

Figure 1 shows examples of ^32^P autoradiographs obtained by testing a blank, a positive control (BPDE-dG), and the lung DNA from Swiss H mice, either untreated (sham-exposed) or exposed to MCS during the first 10 weeks of life, and either untreated or treated with oral celecoxib for 6 weeks after weanling. Exposure of mice to MCS caused the formation of a massive diagonal radioactive zone (DRZ), which is typical of exposures to complex mixtures. Administration of celecoxib to MCS-free mice resulted in the formation of 3 autoradiographic spots that were absent in sham-exposed mice. It is not possible to ascertain whether the same DNA adducts were also present in the lung of MCS-exposed mice treated with this drug because the autoradiographic spots may have been masked and overwhelmed by the MCS-induced DRZ.

Table 1 (left column) reports the levels of pulmonary bulky DNA adducts in the 4 experimental groups, each one composed of 5 males (M) and 5 females (F). There were no significant intergender differences within any groups. Treatment of mice with celecoxib caused a significant, 3.3-fold increase of DNA adducts, as compared with sham-exposed mice, which reflects the presence of the 3 celecoxib-specific autoradiographic spots shown in Figure 1. The autoradiographic DRZ detected by ^32^P postlabeling in MCS-exposed mice corresponded to a 14.5-fold increase of pulmonary DNA adduct levels in combined genders. Administration of celecoxib to MCS-exposed mice significantly decreased the formation of DNA adducts in parallel to an evident attenuation of the DRZ (see Figure 1). In any case, DNA adduct levels in this group were still significantly higher (8.5-fold) compared to sham-exposed mice.

**Table 1:** Bulky DNA adducts and 8-oxo-dGuo evaluated by ^32^P postlabeling in the lungs of 40 Swiss H mice belonging to 4 experimental groups, as related to gender, exposure to MCS, and treatment with celecoxib

Treatment	Gender	Adducts/ 10^8^ nucleotides	8-oxo-dGuo/ 10^5^ nucleotides
Sham	M	1.3 ± 0.19	1.7 ± 0.25
	F	1.2 ± 0.17	2.1 ± 0.20
	M + F	1.3 ± 0.12	1.9 ± 0.17
Celecoxib	M	4.3 ± 0.40^b^	1.6 ± 0.29
	F	4.3 ± 0.34^b^	2.3 ± 0.28
	M + F	4.3 ± 0.25^b^	1.9 ± 0.22
MCS	M	17.9 ± 1.77^b^	5.0 ± 0.18^b^
	F	20.0 ± 3.30^b^	6.0 ± 0.44^b^
	M + F	18.9 ± 1.59^b^	5.5 ± 0.26
MCS + Celecoxib	M	11.5 ± 2.08^b,c^	2.2 ± 0.27^c^
	F	9.8 ± 1.91^a,c^	1.9 ± 0.22^c^
	M + F	11.0 ± 0.30^b,c^	2.1 ± 0.17^c^

The results are means ± SE of the results obtained in the 5 mice composing each group.

Statistical analysis: ^a^*P* < 0.01 and ^b^*P* < 0.001, as compared with sham-exposed mice; ^c^*P* < 0.05 and ^d^*P* < 0.001, as compared with MCS-exposed mice.

### Oxidative DNA damage in the lung

Table 1 (right column) reports the levels of pulmonary 8-oxo-dGuo in the DNA of the same 4 experimental groups of Swiss H mice used for measuring bulky DNA adducts. Again, there was no intergender differences within any group. Exposure of mice to MCS caused a significant oxidative DNA damage in the lung, as documented by a 2.9-fold, statistically significant increase of 8-oxo-dGuo levels. Administration of celecoxib to MCS-free mice did not affect 8-oxo-dGuo levels as compared with sham-exposed mice, whereas its administration to MCS-exposed mice significantly attenuated the oxidative DNA damage to such an extent that the 8-oxo-dGuo levels detected in MCS-exposed mice treated with celecoxib were undistinguishable from those detected in sham-exposed mice.

### Systemic MCS genotoxicity

Modulation by celecoxib of the systemic genotoxicity was evaluated by measuring the frequency of MN NCE in the blood of mice exposed to MCS during the first 4 months of life, since birth, and/or treated with celecoxib after weanling until the 4th month of life. Exposure to MCS of both male and female mice resulted in significant increases in MN NCE frequency, as compared with sham-exposed mice (1.9 ± 0.14 *vs.* 1.3 ± 0.10 in males and 1.2 ± 0.06 *vs.* 0.8 ± 0.08 in females; *P <* 0.01 in both cases). Administration of celecoxib had no significant effect on the frequency of MN NCE either in MCS-free mice (1.4 ± 0.12 in males and 0.8 ± 0.08 in females) or in MCS-exposed mice (2.2 ± 0.15 in males and 1.2 ± 0.08 in females).

### Modulation of pulmonary miRNAs

**Figure 2:**
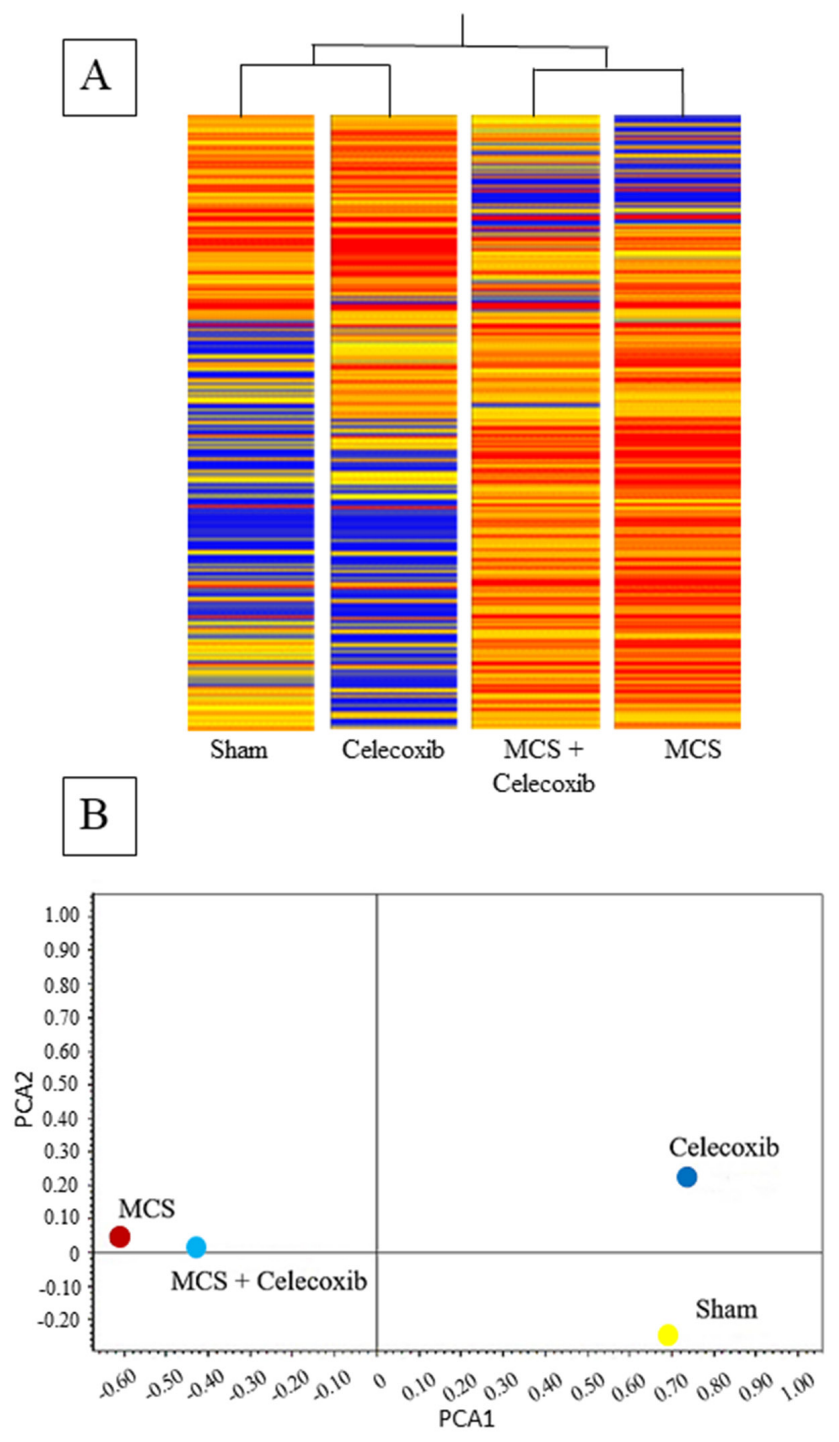
HCA **(A)** and bidimensional PCA **(B)** comparing the expression profiles of pulmonary miRNAs in sham-exposed mice and mice exposed to MCS during the first 10 weeks of life, either untreated or treated with celecoxib for 6 weeks after weanling.

miRNA expression profiles were analyzed in the Swiss H mice belonging to the same 4 experimental groups examined for DNA end-points. Overall comparisons are shown in Figure 2, which reports the results of HCA (A) and bidimensional PCA (B). According to the former approach, it appears that Sham and MCS are allocated far away in the dendrogram. Celecoxib is close to Sham, and MCS + celecoxib is linked to the MCS branch. Similarly, the Sham and MCS symbols fall in opposite quadrants at PCA. Celecoxib is in the quadrant nearby Sham, and MCS + Celecoxib is close to MCS.

**Figure 3:**
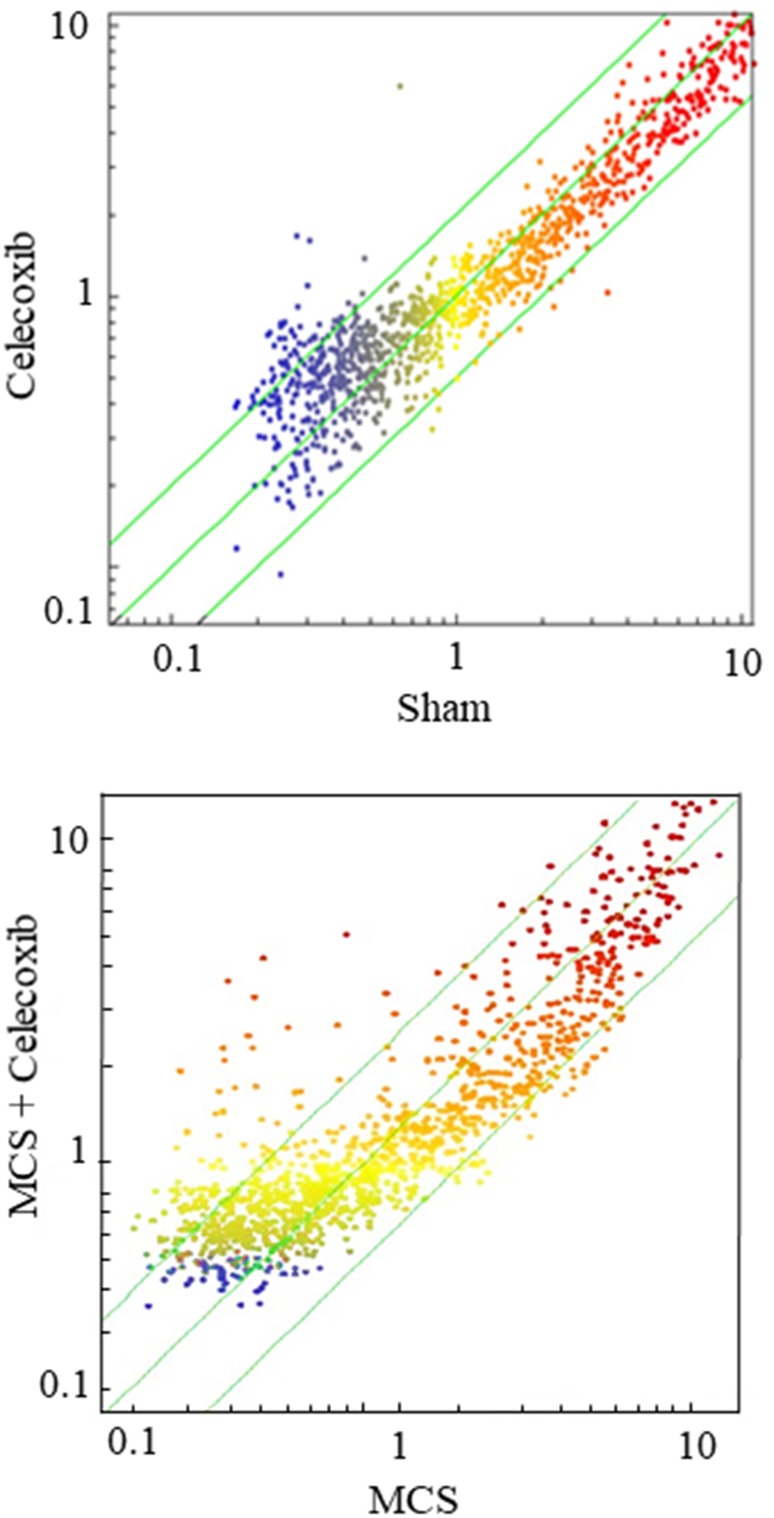
SPAs comparing the expression of 1,135 pulmonary miRNAs either in sham-exposed mice *vs.* mice receiving celecoxib with the diet for 6 weeks, starting after weanling (upper panel), or in mice exposed to MCS during the first 10 weeks of life, in the absence of celecoxib, *vs.* MCS-exposed mice receiving celecoxib (bottom panel) Each dot represents a miRNA, whose expression intensity can be inferred from the position in the x- and y-axes according to a color scale (blue, low; yellow, medium; orange to red, high). The diagonal belts indicate the 2-fold variation intervals. Symbols falling in the upper left area denote miRNA upregulation by celecoxib, and those falling in the bottom right area denote miRNA downregulation by celecoxib.

Figure 3 shows SPAs comparing the intensity of expression of pulmonary miRNAs either in sham-exposed mice *vs.* mice receiving celecoxib (upper panel), or in mice exposed to MCS, in the absence of celecoxib, *vs.* MCS-exposed mice receiving celecoxib (bottom panel). The miRNAs falling outside the diagonal 2-fold were mainly located in the lower part of the diagram, which includes miRNAs expressed at low intensity levels. As inferred from volcano-plot analyses (not shown), the number of miRNAs changing their expression more than 2-fold and above the statistical significance threshold as compared with sham-exposed mice was 21 (1.9%) in celecoxib-treated mice, of which 19 were upregulated and 2 were downregulated. The identity of these miRNAs and their main functions are reported in Table 2 (left column). The right column of the same table reports the miRNAs that were significantly dysregulated by celecoxib in MCS-exposed mice. These included 11 miRNAs (1.0%), all of them upregulated by celecoxib both in MCS-free and MCS-exposed mice.

**Table 2:** List and functions of miRNAs, as inferred from volcano plot analyses, whose expression varied at least 2-fold and to a statistically significant extent in the lung of Swiss H mice as a result of celecoxib administration either to sham-exposed mice or to MCS-exposed mice

miRNA	Celecoxib/Sham	MCS + Celecoxib/MCS	Function
miR-106a	3.27	2.49	Cell adhesion, TNF activation, stress response
miR-181c	2.13	-	NFkB stress response
miR-193	2.77	-	Signal transduction
miR-196b	2.04	-	TGF-beta
miR-208	2.22	-	Heart damage
miR-290	2.21	2.82	Stem cell marker
miR-381	2.66	2.15	Cell proliferation
miR-471	2.97	2.42	LC3-associated phagocytosis
miR-511	2.28	-	Inflammation, monocyte activation
miR-582	3.36	-	Cell proliferation, apoptosis
miR-615	2.37	2.92	Macrophage activation
miR-701	2.54	-	NA
miR-758	3.28	2.71	Cell proliferation, apoptosis
miR-1193	0.41	-	Cell proliferation, apoptosis
miR-1195	0.40	-	MAP kinase signaling pathway, cytochrome CYP2s1 activity
miR-1247	2.30	2.00	Fibroblasts activation, increase of pro-inflammatory gene expression
miR-1251	2.83	3.17	Signal transduction by targeting IGF1
miR-3070	3.38	2.58	NA
miR-3085	2.27	4.17	Cartilage homeostasis and destruction
miR-3109	2.04	2.03	NA
miR-5617	2.13	-	NA

NA, not available.

### Modulation of blood serum miRNAs

**Figure 4:**
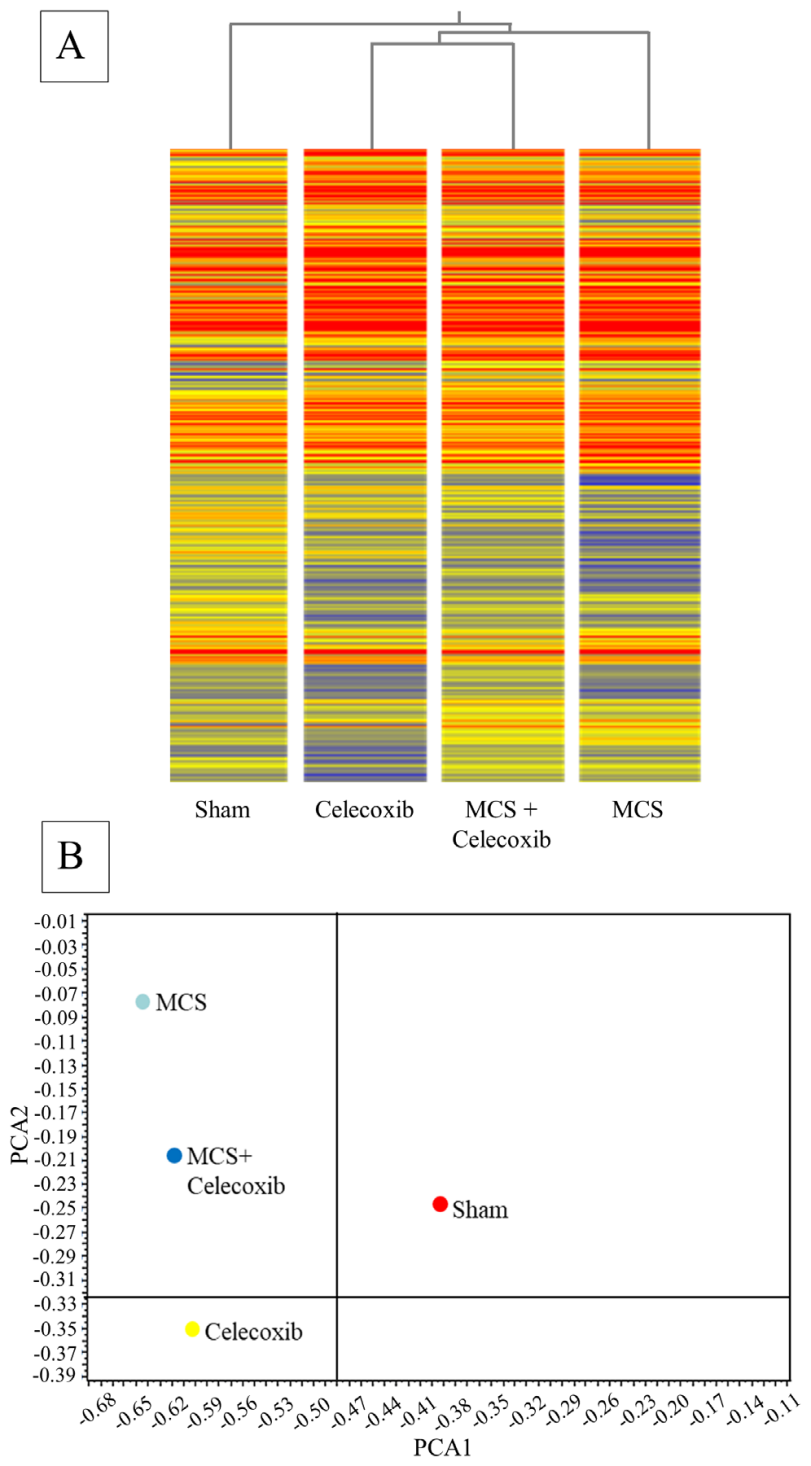
HCA **(A)** and bidimensional PCA **(B)** comparing the expression profiles of miRNAs in the blood serum of sham-exposed mice and MCS-exposed mice, either untreated or treated with celecoxib as described in the legend to Figure 2.

The concentrations of miRNAs in blood serum were evaluated in the same Swiss H mice in which pulmonary miRNAs had been analyzed. The comparison of Figure 2A and Figure 4A shows at a glance that the concentrations of circulating miRNAs were much lower than those of pulmonary miRNAs, as inferred from the different color of the bands composing each column (blue, low intensity; red, high intensity). The distribution patterns of the experimental groups in the hierarchical tree (Figure 4A) shows that Sham and MCS are far away in the dendrogram. Celecoxib and MCS + Celecoxib are close in the same branch, which is linked to the MCS branch. Allocation of symbols according to bidimensional PCA (Figure 4B) shows that MCS and Sham are located far away from each other, with the MCS + Celecoxib symbol half-way between Sham and MCS and the Celecoxib symbol allocated from the same side of Sham.

**Figure 5:**
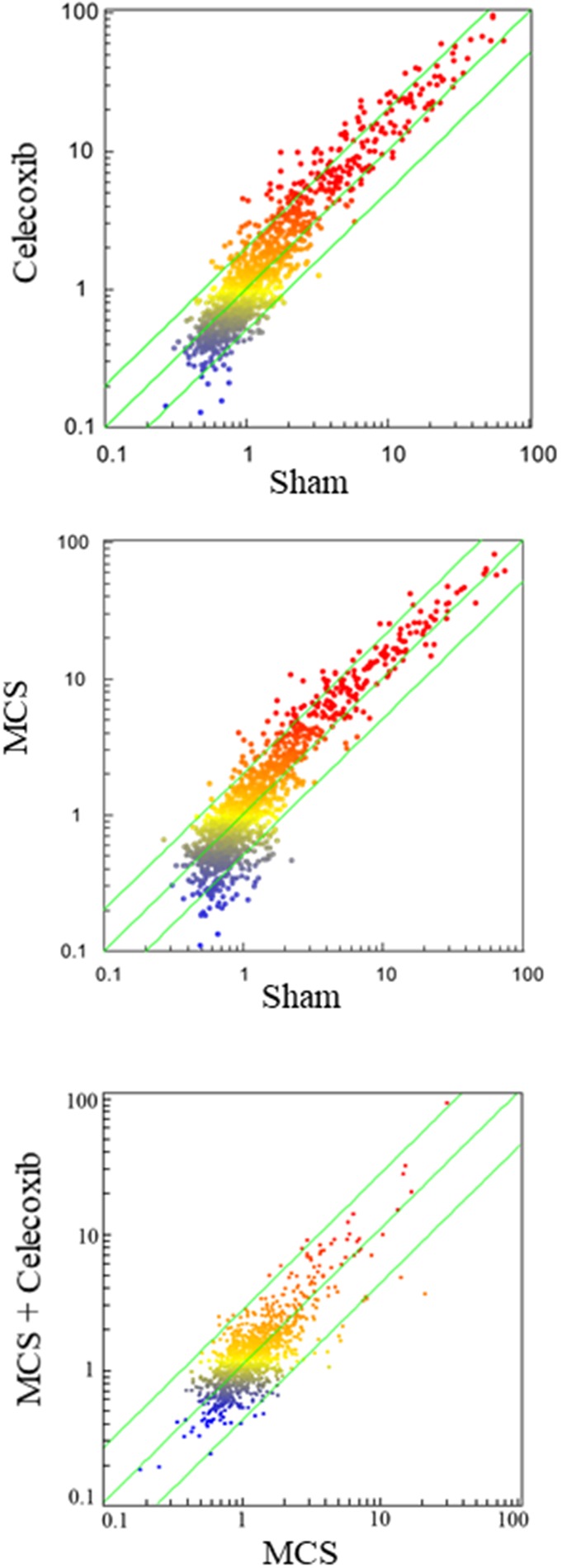
SPAs comparing the expression of 1,135 miRNAs in the blood serum of sham-exposed mice *vs.* either celecoxib-treated mice (upper panel) or MCS-exposed mice (middle panel) or MCS + celecoxib *vs.* mice exposed to MCS in the absence of celecoxib (bottom panel) Each dot represents a miRNA, whose expression intensity can be inferred from the position in the x- and y-axes according to a color scale (blue, low; yellow, medium; orange to red, high). The diagonal belts indicate the 2-fold variation intervals.

SPA compared miRNA levels in the blood serum of sham-exposed mice *vs.* either celecoxib-treated mice (Figure 5, upper panel) or MCS-exposed mice (Figure 5, middle panel) or MCS+celecoxib *vs.* MCS-exposed mice in the absence of chemopreventive agent (Figure 5, bottom panel). The concentration of several miRNAs was altered in the blood serum of celecoxib-treated mice as compared with sham-exposed mice. Identity, variations, and functions (as inferred from literature and Targetscan database) of the 18 miRNAs that were significantly modulated in blood serum by celecoxib, as compared with sham-exposed mice, are reported in Table 3 (left column). These miRNAs were identified by volcano-plot analysis (not shown) using 2-fold variation and *P* < 0.05 significance ANOVA thresholds. Two of these miRNAs (miR-181, miR-208a) were also upregulated by celecoxib in the lung tissue (see Table 2). In addition, the miRNAs altered by celecoxib in blood serum included many miRNAs that were not modulated by this drug in the lung. Seven of the 18 blood miRNAs altered by celecoxib are liver specific, thus their presence in blood reflects celecoxib-induced modifications occurring in this organ. It is of interest that miR-208a, which has been related to the occurrence of heart damage, was well detectable in blood serum following celecoxib administration.

**Table 3:** Fold variations, main functions, and tissue specificity of miRNAs that were significantly modulated in the blood serum of Swiss H mice, either treated with celecoxib (left column) or exposed to MCS (right column), as compared with sham-exposed mice

miRNA	Fold variation			Function	Tissue specificity
	Celecoxib/ Sham	MCS/ Sham	MCS/ MCS+Celecoxib		
miR-9	-	3.12		Apoptosis	Brain
miR-21/miR-21a	2.65	2.25		Tumor suppressor gene *PTEN*, cell proliferation	Lung, kidney, bladder, liver
miR-30a	-	3.02	2.75	Intercellular adhesion, protein repair, NFκB activation, cell cycle, EGF activation, stem cell recruitment, multidrug resistance	Kidney, lung, heart
miR-125b	2.64	1.54		Oncogene *ERBB*, vitamin D receptor, inflammation, gene transcription	Lung, cervix, brain, ovary, prostate, bladder
miR-181/ miR-181b	2.89	2.79		NFκB stress response	Brain, thymus, kidney, lung^a^
miR-184	-	3.44		Cell differentiation	Epithelia, eye, tongue, brain, lung^a^
miR-185	2.70	1.86		Cell proliferation, cholesterol metabolism	Liver, breast, prostate, ovary
miR-208	2.63	-		Heart damage	Heart, lung^a^
miR-211	-	4.35	2.88	Stress response, oncogene (TGF) suppression	Skin, pancreas
miR-222	2.42	1.53		Angiogenesis, cell proliferation	Prostate, lung, bladder
miR-296	2.83	3.05		Thioredoxin and cysteine synthesis (antioxidants), inflammation	Muscle, prostate, lung, bladder
miR-301a	2.60	2.59		Stress response, oncogene activation	Liver, stomach, pancreas, lung^a^
miR-320	-	3.28		Protein repair, intracellular trafficking, cell proliferation	Bladder, cervix, liver, lung
miR-328	2.53	-		Stem cell differentiation	Intestine, heart
miR-329	-	3.22		Cell proliferation, coagulation	Platelet, brain, lung^a^
miR-335	2.40	2.42	2.51	Cell proliferation, apoptosis	Lung
miR-341	-	3.31		Cell differentiation	Lung^a^
miR-361	2.88	1.88		Angiogenesis	Blood vessels
miR-374b	-	3.00	2.63	Stress response	Lung
miR-379	2.92	1.87		Signal transduction, ATP metabolism	Liver
miR-490	2.48	-		Stress response, cell proliferation	Lung
miR-551/ miR-551b	2.43	1.48		DNA repair, inflammation, cell proliferation	Liver
miR-695	2.90	2.27		NA	Lung^a^
miR-802	0.39	-		Glucose metabolism	Liver
miR-804	2.86	2.36		Cell proliferation, collagen production, *Ras* activation	Liver, lung
miR-1199	-	3.13	2.62	NA	Lung^a^
miR-1224	-	3.66		Cell proliferation inhibition, angiogenesis	Liver, blood vessels, lung^a^
miR-1298	-	5.81		Cell proliferation, apoptosis	Brain
miR-1894	-	0.43		Cell proliferation, migration	Breast
miR-1900	-	2.39		Cell homeostasis	NA
miR-1934	-	4.13		Inflammatory response	Adipose tissue
miR-1964	2.61			NA	NA
miR-1983	-	2.38		Aldosterone signaling pathway	Kidney
miR-3068	-	2.56	2.12	NA	NA
miR-3080	-	4.93	2.33	NA	NA

NA, not available. ^a^This study.

MCS was much more effective than celecoxib in altering miRNA expression in blood serum. The SPA reported in Figure 5 (bottom panel) shows that multiple miRNAs fall outside the 2-fold variation interval in MCS-exposed mice as compared with sham-exposed mice. Few miRNAs, mainly expressed at low levels, are downregulated (bottom right area, blue color dots falling outside the 2-fold variation interval). Conversely, the majority of MCS-altered miRNAs were upregulated and characterized by high level of expression (top left area, red dots falling outside the 2-fold variation interval). Identity, variations, and functions of the 30 blood miRNAs that were significantly altered by MCS compared with sham-exposed mice, as identified by volcano-plot analysis (not shown), are reported in Table 3. MCS induced a prevalent upregulation of miRNAs. In fact, as many as 29 out of the 30 altered miRNAs were upregulated.

Celecoxib was effective in counteracting the effect of MCS on 7 blood miRNAs (Table 3). This finding reflects chemopreventive mechanisms activated by this drug in MCS target organs, mainly including the lung but also kidney and skin, as indicated by the tissue specificity of the modulated miRNAs (see Table 3, last column on the right).

### Study 2. Bulky DNA adducts in lung, liver, and heart of ICR (CD-1) mice exposed to MCS and/or treated with celecoxib

**Figure 6:**
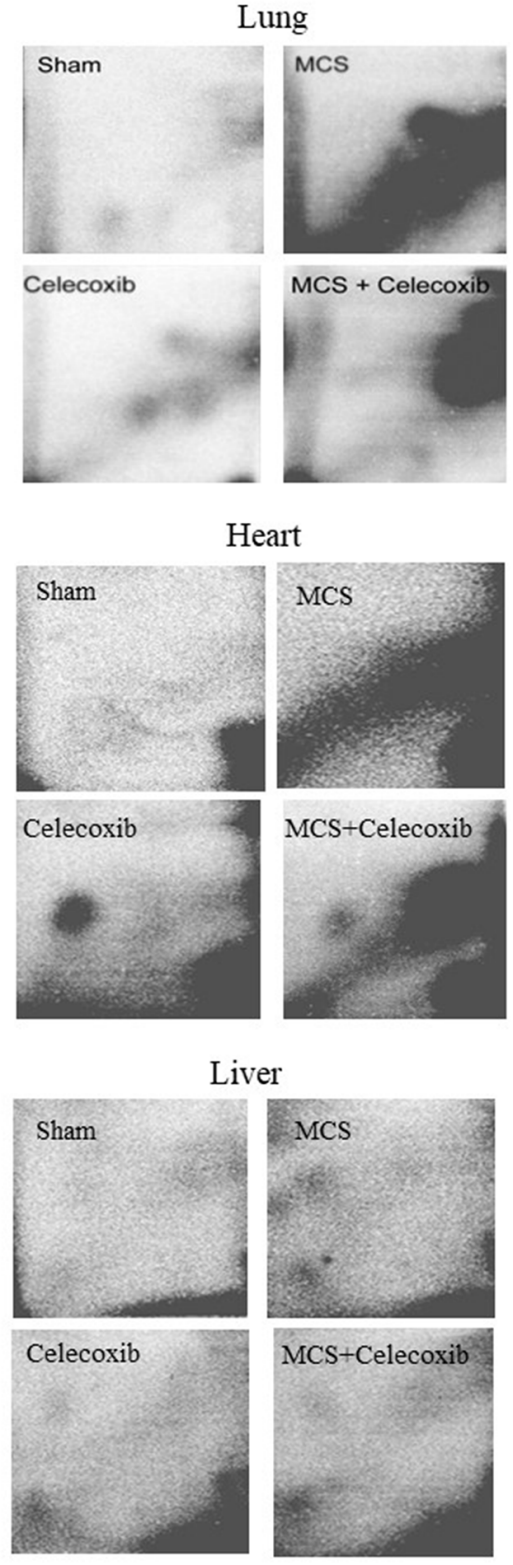
Examples of ^32^P autoradiographs obtained by testing the lung, heart, and liver DNA from ICR (CD-1) mice, either untreated (sham-exposed) or treated with celecoxib for 6 weeks after weanling or exposed to MCS for 10 weeks since birth, in the absence of celecoxib, or exposed to MCS and treated with celecoxib

Figure 6 shows examples of ^32^P autoradiographs obtained by testing the lung, liver, and heart DNA from ICR (CD-1) mice, as related to treatment with celecoxib and/or exposure to MCS. Exposure of mice to MCS resulted in the formation of massive DRZs in both heart and lung, whereas in the liver such an effect was marginal. Treatment of smoke-free mice with celecoxib caused the formation of 3 autoradiographic spots in the lung, having a localization similar to the one observed in the previous study (see Figure 1), and of an evident spot in the heart, which was not discernible in the liver. Administration of celecoxib to MCS-exposed mice resulted in a sharp reduction of DRZs in both lung and heart. While the celecoxib-related spots were no longer detectable in the lung, also because they were partially masked by the DRZ, the same celecoxib-related spot detected in the heart of smoke-free mice was attenuated but still visible in the heart of MCS-exposed mice.

Table 4 reports the levels of bulky DNA adducts (means ± SE of 10 mice per gender) in the three examined organs. In the lung, exposure of mice to MCS caused a considerable and statistically significant increase of DNA adduct levels in both males (10.7-fold) and females (12.1-fold), with an average 11.5-fold increase in combined genders. Likewise, exposure of mice to MCS caused a considerable and statistically significant increase of DNA adduct levels in in the heart of both males (4.6-fold) and females (5.9-fold), with an average 5.1-fold increase in combined genders. The slight differences between males and females were not statistically significant.

**Table 4:** Bulky DNA adducts evaluated by ^32^P postlabeling in the lungs, heart and liver of 80 ICR (CD-1) mice, as related to gender, exposure to MCS, and treatment with celecoxib

Treatment	Gender	Adducts/10^8^ nucleotides		
		Lung	Heart	Liver
Sham	M	1.5 ± 0.18	1.6 ± 0.09	1.2 ± 0.09
	F	1.5 ± 0.20	1.5 ± 0.05	1.2 ± 0.09
	M + F	1.5 ± 0.12	1.6 ± 0.05	1.2 ± 0.06
Celecoxib	M	4.4 ± 0.56^c^	3.3 ± 0.18^c^	1.6 ± 0.12^a^
	F	3.7 ± 0.31^c^	3.6 ± 0.37^c^	1.8 ± 0.08^c^
	M + F	4.0 ± 0.31^c^	3.4 ± 0.17^c^	1.7 ± 0.08^a^
MCS	M	16.1 ± 0.97^c^	7.4 ± 0.54^c^	1.8 ± 0.17^b^
	F	18.2 ± 1.46^c^	8.9 ± 0.73^c^	1.9 ± 0.17^b^
	M + F	17.2 ± 0.87^c^	8.2 ± 0.45^c^	1.9 ± 0.13^c^
MCS + Celecoxib	M	10.5 ± 0.89^c,e^	6.9 ± 0.35^c^	1.6 ± 0.11^a^
	F	10.8 ± 1.09^c,e^	6.1 ± 0.25^c,d^	1.8 ± 0.10^c^
	M + F	10.7 ± 0.69^c,e^	6.4 ± 0.23^c,d^	1.7 ± 0.08^c^

The results are means ± SE of the results obtained in 10 mice per group.

Statistical analysis: ^a^*P* < 0.05, ^b^*P* < 0.01, and ^c^*P* < 0.001, as compared with sham-exposed mice; ^d^ < 0.01 and ^e^*P* < 0.001, as compared with MCS-exposed mice.

Although to a lower extent, being attributable to isolated spots rather than to a DRZ, the increases of DNA adduct levels in smoke-free mice treated with celecoxib were evident and statistically significant both in the lung (2.9-fold in males, 2.5-fold in females, and 2.7-fold in combined genders) and in the heart (2.1-fold in males, 2.4-fold in females, and 2.2-fold in combined genders). On the other hand, administration of celecoxib significantly attenuated DNA adduct levels in the lung of MCS-exposed mice (1.5-fold decrease in males, 1.7-fold decrease in males, and 1.6-fold decrease in combined genders as compared with MCS-exposed mice in the absence of celecoxib). A similar protective effect was detected in the heart of MCS-exposed mice, an effect that was not statistically significant in males (1.1-fold decrease) but reached the statistically significant threshold in both females (1.5-fold decrease) and combined genders (1.3-fold decrease). In any case, DNA adduct levels in both lung and heart of MCS-exposed mice treated with celecoxib were still much higher than those detected in sham-exposed mice. In the liver, the levels of DNA adducts in mice treated with either MCS and/or celecoxib varied to a statistically significant extent but the differences were very modest and of the same order of magnitude for all treatments. In fact, the increases over sham-exposed mice were 1.5-fold in males, 1.6-fold in both females and combined genders of MCS-exposed mice in the absence of celecoxib; 1.3-fold in males, 1.5-fold in females, and 1.4-fold in combined genders of MCS-free mice treated with celecoxib; and 1.3-fold in males, 1.5-fold in females, and 1.4-fold in mixed genders of MCS-exposed mice treated with celecoxib.

### Study 3. Modulation by celecoxib of miRNA expression profiles in 10 organs and 3 body fluids of young Swiss ICR (CD-1) mice, either smoke-free or MCS-exposed

#### Survival and body weights

All mice survived after 8 weeks of treatment. The body weights in sham-exposed mice at the beginning of the study were 38.3 ± 0.83 in males and 28.8 ± 0.82 in females and grew progressively until reaching a plateau after 3 weeks. After 8 weeks, the body weights were 42.3 ± 1.09 in males and 37.5 ± 1.16 in females. From the 3rd week onwards, exposure to MCS resulted in a slight but statistically significant loss of body weight, which after 8 weeks was 39.4 ± 0.70 in males (*P <* 0.05 as compared to sham) and 31.7 ± 1.22 in females (*P <* 0.01).

As compared with sham-exposed mice, administration of celecoxib at high dose (1,600 mg/kg diet) did not significantly affect the body weight, which was 42.1 ± 0.61 in males and 31.5 ± 0.98 in females. Likewise, administration of celecoxib at low dose (20 mg/kg diet) to smoke-free female mice did not significantly affect the body weight, which was 32.6 ± 1.82. The slight body weight loss caused by MCS was not significantly affected by administration of celecoxib either at high dose (34.6 ± 1.55 in males and 26.7 ± 0.56 in females) or at low dose (30.9 ± 1.24 in females).

### Effect of high dose celecoxib on miRNA expression in 10 organs and 3 body fluids of mice, either sham-exposed or MCS-exposed

**Figure 7:**
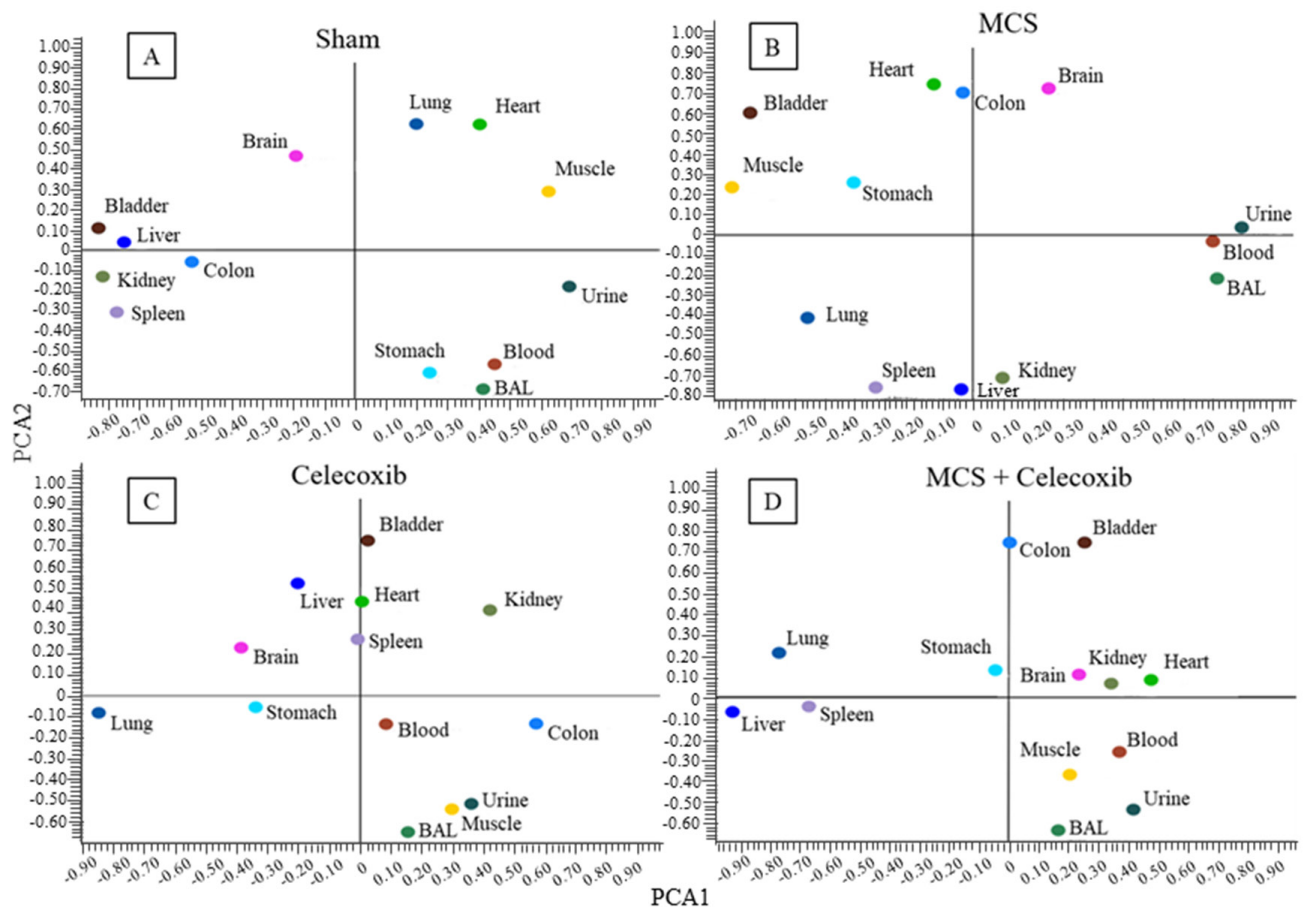
Bidimensional PCA comparing the expression profiles of miRNAs in 10 organs and 3 body fluids of adult ICR (CD-1) mice, either sham-exposed **(A)** or exposed to MCS for 8 weeks **(B)**, and either administered celecoxib to smoke-free mice **(C)** or MCS-exposed mice **(D)**.

Administration of high dose celecoxib (1,600 mg/kg diet) altered the position of miRNA profiles as compared to sham-exposed mice, as evaluated by bidimensional PCA (Figure 7C *vs.* Figure 7A). The organs changing the PCA quadrant compared to Sham-treated mice as a consequence of celecoxib administration were the stomach, colon, spleen, kidney, lung, and skeletal muscle. The influence of celecoxib on miRNA expression in body fluids was less remarkable. The organs changing PCA quadrant as a consequence of celecoxib administration to MCS-exposed mice (Figure 7D, as compared to MCS-exposed mice in the absence of the drug (Figure 7B), were lung, kidney, bladder, and muscle. The effects of celecoxib were also detectable in body fluids of MCS-exposed mice, especially in urine and BALF.

**Figure 8:**
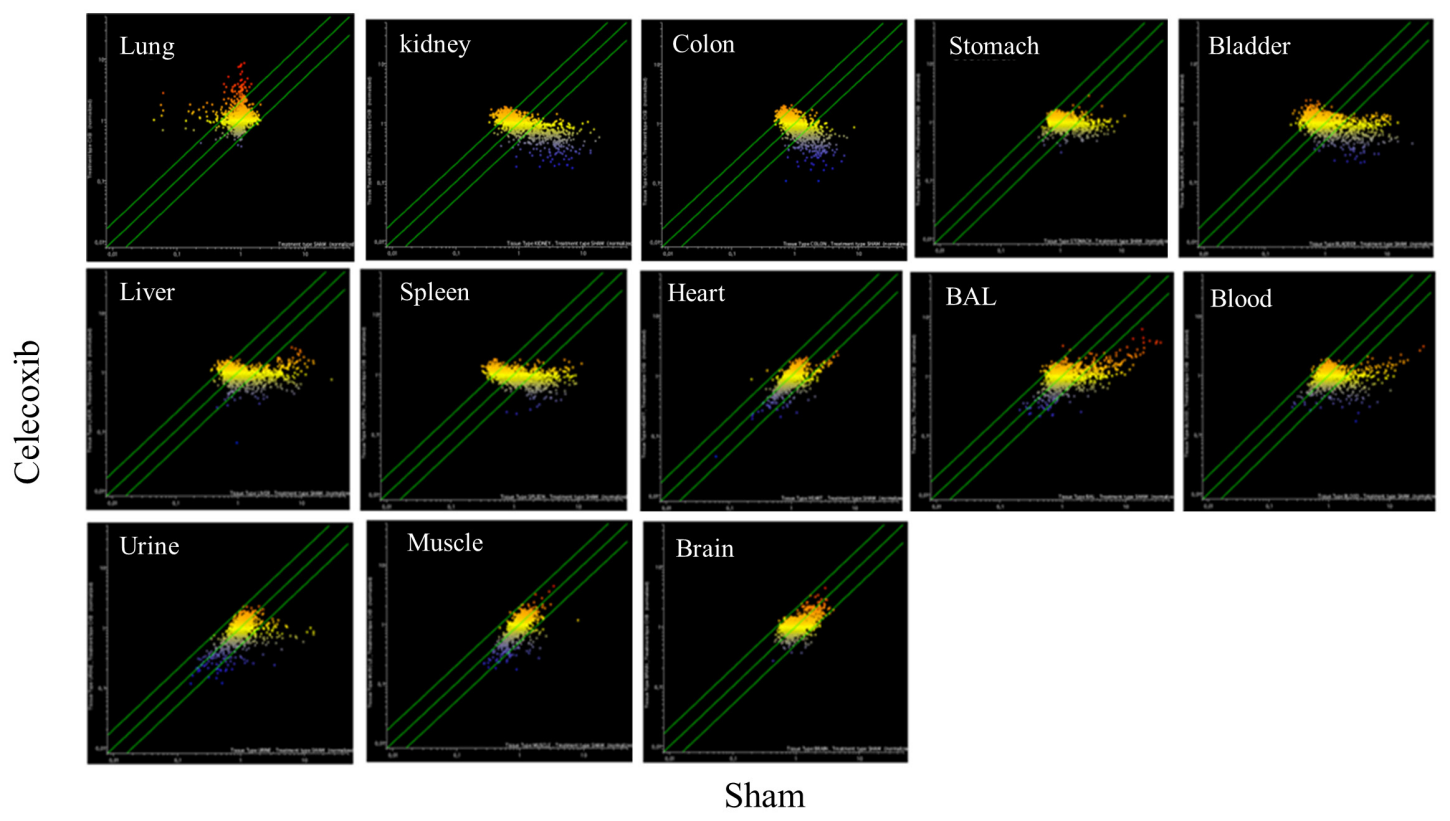
SPAs comparing the expression of 1,135 pulmonary miRNAs in 10 organs and 3 body fluids of ICR (CD-1) mice in celecoxib-treated *vs.* sham-exposed mice Each dot represents a miRNA, whose expression intensity can be inferred from the position in the x- and y-axes according to a color scale (blue, low; yellow, medium; orange to red, high). The diagonal belts indicate the 2-fold variation intervals. Symbols falling in the upper left area denote miRNA upregulation by celecoxib, and those falling in the bottom right area denote miRNA downregulation by celecoxib.

**Figure 9:**
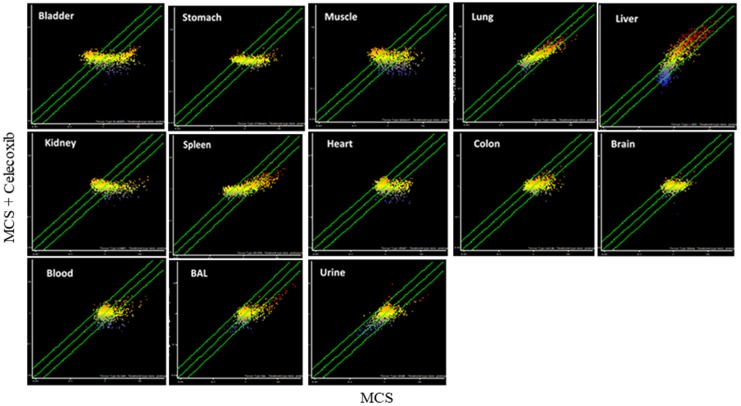
SPAs comparing the expression of 1,135 pulmonary miRNAs in 10 organs and 3 body fluids of ICR (CD-1) mice, either MCS-exposed and treated with celecoxib or MCS-exposed in the absence of celecoxib Each dot represents a miRNA, whose expression intensity can be inferred from the position in the x- and y-axes according to a color scale (blue, low; yellow, medium; orange to red, high). The diagonal belts indicate the 2-fold variation intervals. Symbols falling in the upper left area denote miRNA upregulation by celecoxib, and those falling in the bottom right area denote miRNA downregulation by celecoxib.

The celecoxib organotropism was evaluated with more details by comparative SPA (Figure 8). According to the number of miRNAs varying their expression more than 2-fold as a consequence of celecoxib treatment (i.e., being located outside the diagonal area limited by green lines in Figure 8), it appears that the preferentially targeted organs were lung and bladder. An intermediate effect was detected in liver, kidney, spleen, colon, and stomach; only minimal effects were observed in heart, skeletal muscle, and brain. miRNA expression in body fluids was markedly affected by celecoxib treatment, especially in blood and BAL, and remarkable effects were also detected in urine. Figure 9 shows the overall protective effects of celecoxib from MCS as evaluated by SPA. According to the number of miRNAs varying their expression more than 2-fold as a consequence of celecoxib administration to MCS-exposed mice (i.e., being located outside the diagonal area limited by green lines in Figure 9), the preferentially protected organs by celecoxib were bladder, kidney, and spleen; intermediate effects were detectable in stomach, colon, skeletal muscle, heart, liver, and lung; no effect was detectable in the brain. As to body fluids, blood and BAL were more sensitive than urine in detecting the protective effects of celecoxib against miRNA alteration induced by MCS.

Table 5 provides a list reporting the identity and functions of miRNAs modulated by celecoxib in 9 organs and 3 body fluids of ICR (CD-1) mice, either sham-exposed (superscripts a) or MCS-exposed (superscripts b). The brain is not reported in that table because no brain miRNA was significantly modulated by celecoxib. As inferred from Table 5, 30 miRNAs were modulated by celecoxib in the urinary bladder (upregulated/downregulated in MCS-free mice: 0/10; upregulated/downregulated in MCS-exposed mice: 8/12); 27 in kidney (7/20; 0/0); 20 in stomach (2/12; 3/3); 18 in liver (0/15; 2/1); 18 in blood serum (2/11; 2/3); 17 in spleen (2/7; 1/7); 16 in skeletal muscle (0/0; 6/10); 15 in lung (14/0; 0/1); 7 in colon (1/4; 0/2) and in BALF (2/3; 0/2); 3 in heart (0/2; 0/1) and urines (0/3; 0/0); and 0 in brain.

**Table 5:** MiRNAs modulated by high dose celecoxib (1,600 mg/Kg diet) in 9 organs and 3 body fluids of female ICR (CD-1) mice, either sham-exposed or MCS-exposed

miRNA	Lung	Liver	Heart	Kidney	Spleen	Urinary bladder	Skeletal muscle	Colon	Stomach	Blood serum	BALF	Urine	Main functions
miR-21a									0.39^a^	0.27^a^			Tumor suppressor gene PTEN, cell proliferation
miR-30a	2.04^a^												Intercellular adhesion, protein repair, NFkB activation, cell cycle, EGF activation, stem cell recruitment, multidrug resistance
miR-30b		0.26^a^	0.41*^a^* 0.36^b^	0.25*^a^*	0.31^b^	0.22^b^		0.23*^a^*					Intercellular adhesion, protein repair, NFkB activation, cell cycle, EGF activation, stem cell recruitment, multidrug resistance
miR-30c-1-3p						0.33^b^			0.25^a^				Intercellular adhesion, protein repair, NFkB activation, cell cycle, EGF activation, stem cell recruitment, multidrug resistance
miR-30c-2-3p		0.11^a^		0.07^a^	0.30^b^	0.14^b^							Intercellular adhesion, protein repair, NFkB activation, cell cycle, EGF activation, stem cell recruitment, multidrug resistance
miR-30c-5p			0.46^a^	0.08^a^	0.15^a^	0.10^a^	0.23^b^	0.33^b^	0.40^a^	0.26^a*^	0.26^a^		Intercellular adhesion, protein repair, NFkB activation, cell cycle, EGF activation, stem cell recruitment, multidrug resistance
miR-30d	2.64^a^						2.58^b^						Intercellular adhesion, protein repair, NFkB activation, cell cycle, EGF activation, stem cell recruitment, multidrug resistance
miR-30e	3.10^a^	0.10^a^		0.12^a^		0.13^b^ 0.12^a^	0.11^b^			0.28^a^			Intercellular adhesion, protein repair, NFkB activation, cell cycle, EGF activation, stem cell recruitment, multidrug resistance
miR-106a							2.65^b^			2.03^a^			Cell adhesion, TNF activation, stress response
miR-125a		0.35^a^ 2.08^b^		0.31^a^	0.49^a^		0.22^b^		0.32^a^ 0.35^b^				Oncogene ERBB, vitamin D receptor, inflammation, gene transcription
miR-125b	2.16^a^			0.23^a^	0.29^a^	0.42^b^	0.09^b^		0.28^a^ 0.11^b^				Oncogene ERBB, vitamin D receptor, inflammation, gene transcription
miR-181b	4.08^a^					2.43^b^			2.60^b^		0.48^a^		NFkB stress response
miR-181c-3p								0.40^a^					NFkB stress response
miR-181c-5p						2.83^b^	4.78^b^						NFkB stress response
miR-181d	2.78^a^					2.73^b^			0.46^a^				NFkB stress response
miR-185-3p				0.39^a^	0.29^b^		0.37^b^		0.29^a^				Cell proliferation, cholesterol metabolism
miR-185-5p	0.47^b*^	0.06^a^		0.04^a^	0.48^b^	0.07^b^ 0.05^a^		0.12^a^		0.38^a*^ 0.35^b^			Cell proliferation, cholesterol metabolism
miR-193b		0.30^b^		2.11^a^									Signal transduction
miR-196a-3p					0.20^a^								TGF-beta
miR-196a-5p		0.25^a^		0.27^a^	0.28^a^	0.18^b^		0.34^a^		2.13^b^			TGF-beta
miR-208a-3p					0.15^a^								Heart damage
miR-208a-5p				0.21^a^		0.18^b^			0.43^a^		0.47^a^	0.45^a^	Heart damage
miR-208b				2.66^a^		0.18^b^			2.02^b^				Heart damage
miR-211									2.30^b^				Stress response, oncogene (TGF) suppression
miR-222								2.76^a^					Angiogenesis, cell proliferation
miR-290										0.15^a^		0.12^a*^	Stem cell marker
miR-296	2.15^a*^												Thioredoxin and cysteine synthesis (antioxidants), Inflammation
miR-301a		0.22^a^		0.14^a^		0.13^a^		0.18^a^					Stress response, oncogene activation
miR-301b		0.30^a^		0.41^a^		0.43^b^ 0.41^a^	0.30^b^						Stress response, oncogene activation
miR-335-3p		0.26^a^		0.24^a^						0.25^b^ 0.22^a^			Cell proliferation, apoptosis
miR-335-5p				0.05^a^		0.07^a^							Cell proliferation, apoptosis
miR-361	8.09^a^	0.33^a^		0.20^a^	0.35^b^		0.34^b^		0.23^a^	0.29^a^			Angiogenesis
miR-374b	2.06^a^			0.48^a^									Cell proliferation, invasion
miR-379						0.42^a^							Signal transduction
miR-381						0.42^b^							Cell proliferation
miR-471	3.72^a^	0.14^a^		2.99^a^	3.20^b^				2.02^b^	0.26^a^			NA
miR-511-3p					0.18^a^								Inflammation, monocyte activation
miR-511-5p		2.06^b^		2.33^a^			4.52^b^		0.43^b^				Inflammation, monocyte activation
miR-551b	3.08^a*^												DNA repair, inflammation, cell proliferation
miR-615				0.24^a^		3.53^b^				0.33^a^			Macrophage activation
miR-695				0.33^a^		0.44^b^	0.17^b^		0.21^a^	0.28^a^	0.17^a^		NA
miR-701-3p					2.78^a^ 2.32^b^	4.84^b^	0.25^b^			0.47^b^	0.30^a^		NA
miR-701-5p					0.30^b^								NA
miR-758												2.17^b^	Cell proliferation, apoptosis
miR-802-3p		0.14^a^		0.16^a^		4.86^b^			2.99^b^				Glucose metabolism
miR-802-5p					0.22^a^								Glucose metabolism
miR-804	3.71^a^								0.23^a^				Cell proliferation, collagen production*, Ras* activation
miR-1195						2.71^b^							MAP kinase signaling pathway, cytochrome CYP2s1 activity
miR-1199						2.66^b^	3.03^b^		0.21^a^				NA
miR-1247	6.08^a^												Fibroblasts activation, increase of pro-inflammatory gene expression
miR-1251				2.63^a^				2.41^b^					Signal transduction by targeting IGF1
miR-1945				2.89^a^							0.45^a^	0.38^a*^	NA
miR-1952								2.14^b^					NA
miR-1964-3p	2.87^a^			2.97^a^									NA
miR-1964-5p				0.15^a^		0.22^b^ 0.14^a^	0.22^b^		0.23^a^	0.13^a^	0.31^b^ 0.17^a^		NA

^*^ Also modulated by low dose celecoxib (20 mg/Kg diet). NA, not available.

The reported values indicate the fold variation of miRNA expression either in celecoxib-treated *vs*. sham-exposed micea or in MCS-exposed mice treated with celecoxib *vs*. MCS-exposed mice in the absence of celecoxibb.

Thus, on the whole upregulation of miRNA expression by celecoxib in smoke-free mice was by far prevalent in the lung, whereas the balance was in favor of downregulation in other organs, such as urinary bladder, kidney, stomach, and spleen, and body fluids (blood serum and urines). In MCS-exposed mice the number of miRNAs modulated by celecoxib was lower as compared with MCS-free mice, with the exception of the urinary bladder and skeletal muscle, in which downregulation was prevailing.

Several miRNAs were upregulated in organs of MCS-free mice while being downregulated in the same organs of MCS-exposed mice, which reflects the ability of celecoxib to alter the physiological miRNA expression but, at the same time, to counteract MCS-related alterations. In addition, a number of miRNAs, often clustered in miRNA families, were modulated by celecoxib in multiple targets, sometimes with high fold variations. For instance, members of the miR-30 family were extensively downregulated by celecoxib. In fact, this drug downregulated miR-30b in the liver, heart, kidney and colon of MCS-free mice as well as in the heart, spleen, and urinary bladder of MCS-exposed mice; miR-30c-2-3p was downregulated by celecoxib in the liver (9-fold) and kidney (14-fold) of MCS-free mice and in the spleen and urinary bladder (7-fold) of MCS-exposed mice; miR-30c-2-5p was downregulated in the heart, kidney (13-fold), spleen (7-fold), urinary bladder (10-fold), stomach, blood serum and BALF of MCS-free mice and in the skeletal muscle and colon of MCS-exposed mice; miR-30e was downregulated in the liver (10-fold), kidney (8-fold), urinary bladder (8-fold), and blood serum of MCS-free mice, whereas it was upregulated in the lung, urinary bladder (6-fold) and skeletal muscle (9-fold) of MCS-exposed mice. Within the miR-125 family, celecoxib downregulated miR-125a in the liver, kidney, spleen, and stomach of MCS-free mice and in the skeletal muscle and stomach of MCS-exposed mice, in which this miRNA was upregulated in the liver; miR-125b was downregulated by celecoxib in the lung, kidney, spleen and stomach of MCS-free mice and in the urinary blood, skeletal muscle (11-fold) and stomach (9-fold) of MCS-exposed mice. Within the miR-185 family, celecoxib downregulated miR-185-3p in the kidney and stomach of MCS-free mice and in the spleen and skeletal muscle of MCS-exposed mice; miR-185-5p was downregulated in the liver, kidney (as much as 25-fold), urinary bladder (20-fold), colon (8-fold) and blood serum of MCS-free mice and in the lung, spleen, urinary bladder (14-fold) and blood serum of MCS-exposed mice. Within the miR-301 family, celecoxib downregulated miR-301 in the liver, kidney (7-fold), urinary bladder (8-fold) and colon (6-fold) of MCS-free mice and miR-301b in the liver, kidney, urinary bladder and skeletal muscle of MCS-free mice and in the urinary bladder of MCS-exposed mice. Furthermore, celecoxib downregulated miR-196a-5p in the liver, kidney, spleen, urinary bladder and colon of MCS-free mice, whereas the levels of the same miRNA were increased in blood serum. Celecoxib downregulated miR-208a-5p in the kidney, stomach, BALF and urines of MCS-free mice and in the urinary bladder of MCS-exposed mice. It caused a strong upregulation of miR-361 in the lung (8-fold), whereas it downregulated this miRNA in the liver, kidney, stomach and blood serum of MCS-free mice as well as in the spleen and skeletal muscle of MCS-exposed mice. miR-511 was upregulated by celecoxib in the kidney of MCS-free mice and in the liver an skeletal muscle (5-fold) of MCS-exposed mice, in which this miRNA was downregulated in the stomach. miR-695 was downregulated in the kidney, stomach, blood serum and BALF of MCS-free mice and in the urinary bladder and skeletal muscle (6-fold) of MCS-exposed mice. miR-701-3p was upregulated in the spleen and downregulated in the BALF of MCS-free mice, whereas it was upregulated in the spleen and urinary bladder (5-fold) and downregulated in the skeletal muscle and blood serum of MCS-exposed mice. miR-802-3p was downregulated in the liver (7-fold) and kidney (6-fold) of MCS-free mice and upregulated in the urinary bladder (5-fold) and stomach (3-fold) of MCS-exposed mice. miR-1945 was upregulated in the kidney, whereas its levels decreased in both BALF and urines of MCS-free mice. miR-1964-5p was downregulated by celecoxib in the kidney (7-fold), stomach (4-fold), blood serum (8-fold) and BALF (6-fold) of MCS-free mice as well as in the urinary bladder (5-fold), skeletal muscle (5-fold) and BALF (3-fold) of MCS-exposed mice.

### Effect of low dose celecoxib on miRNA expression in 10 organs and 3 body fluids of mice, either sham-exposed or MCS-exposed

The effects of low dose celecoxib (20 mg/kg diet) were evaluated in the lung, blood serum, and urines of female mice, either smoke-free or MCS-exposed. In all body compartments, the number of miRNAs modulated by celecoxib when administered at low dose was by far lower than the number observed at high dose, both in the absence and in the presence of MCS exposure. It is of interest that the same pulmonary miRNAs that were modulated by celecoxib at low dose (identified with an asterisk in Table 5) were also modulated by celecoxib at high dose, thus being identifiable as sensitive and specific biomarkers of celecoxib effect in lung.

In particular, in lung low dose celecoxib modulated the expression of miR-296 (2.4-fold upregulation) and miR-551b (2.2-fold upregulation) as compared to sham, and it downregulated the expression of miR185 (2.8-fold downregulation) as compared to MCS-exposed mice. Accordingly, the number of miRNAs modulated by celecoxib at low dose was only 3 as compared to the 14 miRNAs modulated in lung by celecoxib at high dose (see Table 5). In blood serum, low dose celecoxib modulated the expressions of miR30c-5p (2.9-fold downregulation), as compared to sham, and of miR-185 (2.1-fold downregulation) as compared to MCS-exposed mice. Accordingly, the number of miRNAs modulated by celecoxib at low dose was only 2 as compared to the 11 miRNAs modulated by celecoxib at high dose in the blood (see Table 5). In urine, low dose celecoxib modulated the expression of both miR-290 (2.4-fold downregulation) and miR-1945b (2.4-fold downregulation) as compared to sham. Accordingly, the number of miRNAs modulated by celecoxib at low dose in urine was 2 as compared to 3 miRNAs modulated by celecoxib at high dose (see Table 5).

## DISCUSSION

The present article reports the results of three consecutive studies evaluating the effects of treatment with celecoxib and/or exposure to MCS on genomic and miRNA alterations in the lung and blood serum of newborn Swiss H mice (*Study 1*), occurrence of bulky DNA adducts in the lung, liver, and heart of newborn ICR (CD-1) mice (*Study 2*), and alterations of miRNA expression profiles in 10 organs and 3 body fluids of young Swiss ICR (CD-1) mice (*Study 3*). The overall results obtained demonstrate that this NSAID causes genomic and epigenetic alterations in the lung, heart, and other body compartments of MCS-free mice but, on the other hand, its administration exerts protective effects towards imbalances of the same end-points in smoking mice.

In the interpretation of the results obtained it should be kept in mind that in *Study 3* administration of celecoxib started short before beginning of exposure to MCS, whereas in both *Study 1* and *Study 2* administration of celecoxib started about one month after beginning of exposure to MCS, as soon as the mice became able to eat autonomously. In *Study 1* and *Study 2* the mice were treated with celecoxib during the 6 weeks following weanling, i.e., from the 5th to the 10th week of life. This period partially overlaps with adolescence [23], leading to sexual maturity and early adulthood. Note that, besides being prescribed in adults, celecoxib can be also used to treat juvenile rheumatoid arthritis in children 2 years and older (https://www.accessdata.fda.gov/drugsatfda_docs/label/2011/020998s033,021156s003lbl.pdf).

The mice were exposed whole-body to MCS, whereas in humans MCS is inhaled as an undiluted complex mixture directly into the respiratory tract and therefore its doses are probably even higher than those inhaled by mice. The celecoxib dose used (1,600 mg/kg diet) was selected based on the results of a preliminary subchronic toxicity study in which administration of the drug to smoke-free, post-weanling mice for 6 weeks did not cause any body weight or behavioral alterations [18]. Although this dose is rather high, it is similar or even lower than those used in most animal studies available in the literature. Furthermore, it is of the same order of magnitude as the pharmacological doses used in humans. In fact, it has been reported that 1,000 mg/kg diet of celecoxib results in a plasma concentration of 1.6 μg/ml in mice, which approximates the reported therapeutic plasma concentration of celecoxib in humans [6]. In this comparison, it should be taken in mind that, while celecoxib in humans is administered once or twice per day, mice eat continuously depending on their metabolic needs, which results in a highly fractionated drug intake with the diet.

Exposure of both Swiss H mice and ICR (CD-1) mice to MCS during the first 10 weeks of life resulted in the massive formation of bulky DNA adducts to lung DNA, which was documented by autoradiographic DRZs. These patterns are typical of exposures to complex mixtures such as CS [24]. Later on, exposure of H mice to MCS during the first 4 months of life caused a systemic genomic damage, which was demonstrated by a significant increase of micronuclei in the erythrocytes. The contribution of oxidative mechanisms to the formation of MCS-related nucleotide alterations was supported by the parallel sharp increase of 8-oxo-dGuo in mouse lung in Swiss H mice. Likewise, high levels of DNA adducts were detected in the heart of MCS-exposed ICR (CD-1) mice, whereas just a marginal increase was appreciable in their liver. These findings are in agreement with the results of previous studies in adult rats exposed to ECS. The main difference between lung and heart is that DNA adduct levels rapidly decline in the lung upon discontinuation of exposure to CS, whereas they are more persistent in the heart, presumably due to a lower efficiency of DNA repair mechanisms [25]. Cell proliferation is another distinctive mechanism between lung and heart. In fact, the cells of the bronchoalveolar tract are characterized by a high proliferation rate, while cardiomyocytes are perennial cells after birth. Accordingly, while the presence of nucleotide alterations in the lung are predictive of a possible evolution towards cancer, no such evolution can be envisaged in the heart, because cell proliferation is a *condicio sine qua non* for the formation of neoplastic diseases. Rather, nucleotide alterations in cardiomyocytes are possibly related to an evolution towards genuinely degenerative heart diseases [26, 27].

A rather unexpected finding was that treatment with celecoxib caused the evident formation of DNA adducts in both mouse lungs and heart. In fact, the oral administration of this NSAID after weanling, from the 5th to the 10th week of life, resulted in the formation of autoradiographic spots, which was not accompanied by any oxidative DNA damage. Formation of celecoxib-specific DNA adducts in the lung was first demonstrated in the study using Swiss H mice (*Study 1*). In order to validate this finding, we carried out a further *ad hoc* study (*Study 2*), in which we treated mice belonging to another strain [ICR (CD-1)] according to the same protocol. The results confirmed that administration of celecoxib to smoke-free mice causes the formation of DNA adducts in the lung and, additionally, in the heart, whereas this kind of lesion was negligible in the liver. These findings are presumably related to the distinctive pharmacokinetic and metabolism of this NSAID in different organs. It is known that, in various animal species, the methyl group of celecoxib is first oxidized to a hydroxymethyl metabolite, followed by additional oxidation of the hydroxymethyl group to a carboxylic acid metabolite [28]. Using human liver microsomes, methyl hydroxylation of celecoxib was found to be primarily catalyzed by CYP2C9 [29], which is involved in the metabolism of endogenous compounds, drugs, and procarcinogens, also including CS components [30].

The results of our studies agree with the results of another study [31], in which the levels of adducts formed by ^3^H-B(*a*)P [benzo(*a*)pyrene] in CS-exposed mice were significantly decreased by oral celecoxib in the liver but were significantly increased in the lung. These data are not coincident with ours, because we evaluated all smoke-related DNA adducts and not only those formed by BPDE [benzo(*a*)pyrene diolepoxide]. In addition, as we will discuss below, we found that celecoxib decreases MCS-related DNA adduct levels. Nevertheless, these findings suggest a tissue specificity in the ability of celecoxib to form DNA adducts. Koul *et al.* [31] ascribed these differential patterns to the fact that celecoxib administration results in a significant decrease of GST (glutathione *S*-transferase) activity in the lung but not in the liver, which may justify an impaired detoxification of the celecoxib reactive metabolite(s) in the lung.

On the other hand, celecoxib inhibited the formation of MCS-related DNA adducts in the heart and, even more efficiently, in the lung. This demonstrates that celecoxib exerts protective effects towards induction of nucleotide alterations in MCS-exposed mice and that the overall protective effects resulting from co-treatment of mice with MCS and celecoxib are prevalent as compared with the DNA damage produced by this COX-2 inhibitor when given to MCS-free mice. These results are consistent with the finding that celecoxib is able to exert protective effects in Swiss H mice exposed to MCS. In particular, besides attenuating preneoplastic alterations in the urinary tract, celecoxib decreased the multiplicity of MCS-induced lung adenomas. Moreover, the yield of lung malignant tumors was lower in MCS-exposed mice treated with celecoxib compared to those MCS-exposed mice that did not receive the chemopreventive regimen. However, at the same dose used in the present study, celecoxib became toxic to MCS-exposed mice and some signs of hepatotoxicity were detected in MCS-exposed mice treated with celecoxib [18]. In humans, a case-control study showed that use of selective COX-2 inhibitors, among which celecoxib, reduced the lung cancer risk [12], and a trial of celecoxib in former smokers decreased the bronchial Ki-67 labeling index and reduced lung nodules on computed tomography [13]. In human lung carcinoma H1299 cells, celecoxib suppressed several molecular alterations induced by a CS condensate [32].

In addition to genomic damage, exposure of mice to MCS resulted in multiorgan epigenetic alterations, in the form of dysregulation of miRNA profiles. MCS induced a prevalent downregulation of miRNAs in the lung, in agreement with previous studies in rats [33, 34] and mice [35–43]. The same conclusion was drawn in studies evaluating miRNA expression in the bronchial epithelium from humans who smoke [44]. The results relative to dysregulation of miRNA expression in the lung and blood of newborn Swiss H mice (*Study 1*) and in 10 organs and 3 body fluids of young Swiss ICR (CD-1) mice (*Study 3*) have been separately reported and commented in previous studies in which microarray data had been validated by qPCR analyses [22, 42].

Here we will discuss the results relative to dysregulation of miRNA expression following administration of celecoxib either to smoke-free mice or to MCS-exposed mice. In Swiss H mice treated with celecoxib after weanling (*Study 1*), the effects of the drug in the lung was mainly in the sense of upregulation. The most intensely upregulated miRNAs by celecoxib included miR-106b, which is involved in cell adhesion, tumor necrosis factor (TNF) activation, and stress response, and miR-582 and miR-328, both of which are involved in cell proliferation and apoptosis. Two of the miRNAs upregulated by celecoxib in mouse lung (miR-181, miR-208a) were also upregulated in the blood serum, but most miRNAs whose concentrations were increased in the blood were unaffected in the lung. It is conceivable that celecoxib-related miRNAs in blood serum reflect molecular changes induced by this drug not only in lung but also in other organs bearing relevance for the pharmacodynamic activity of this drug, such as liver and heart. In fact, besides miR-208a, which has been related to the occurrence of heart damage (see below), 7 of the 18 blood miRNAs altered by celecoxib are liver specific. Similar conclusions were drawn by evaluating miRNA dysregulation by celecoxib in 10 organs and 3 body fluids of Swiss ICR (CD-1) mice (*Study 3*). In fact, the drug induced the release of miRNAs into the blood mainly from kidney, stomach, and liver. These organs are established targets of this COX-2 inhibitor for both protective and adverse effects (gastritis and nephrotoxicity). A similar situation occurred in the BALF, although the number of miRNAs altered by celecoxib was by far lower than in blood. These finding indicate that celecoxib, administered with the diet, does not preferentially target the lung, this organ being only a secondary target as compared to kidney, stomach, liver, and spleen. In contrast, it is likely that the miRNAs released into BALF reflect plasma ultrafiltration processes contributing to the production of bronchial mucus. Kidney, stomach and bladder were the main contributors of miRNAs modulated by celecoxib treatment in urine.

The comparison of miRNA profiles in mice treated with celecoxib, either exposed or unexposed to MCS, was investigated in order to assess which biological fluid better predicts the preventive effects of this drug against miRNA alterations induced by MCS. In the blood, the great contribution of kidney and bladder is consistent with the fact that these organs are targets for both celecoxib and MCS. The spleen contribution, being this organ widely populated by immune-competent cells, reflects the specific anti-inflammatory activity of celecoxib against MCS-induced inflammation. The analysis of BALF further supported the fact that lung is just a secondary target for celecoxib as compared to other organs. In urines, the number of modulated miRNAs was low.

Among other miRNAs modulated by celecoxib, it is of interest that miR-21 was downregulated both in the stomach and in the blood of ICR (CD-1) mice treated with this NSAID. This miRNA had been found to be upregulated in cultured human gastric adenocarcinoma cells treated with nicotine, which also stimulated COX-2/prostaglandin E (PGE) signaling via modulation of NF-κB activity [45]. In the present study, the transcription factor NF-κB was targeted by a number of miRNAs in organs and body fluids of celecoxib-treated mice, either smoke-free and/or MCS-exposed. In particular, celecoxib-modulated miRNAs that target NF-κB included members of the miR-30 family, such as miR-30a (lung), miR-30b (liver, heart, kidney, colon, heart, spleen, urinary bladder), miR-30c-1-3p (stomach, urinary bladder), miR-30c-2-3p (lung, kidney, spleen, urinary bladder), miR-30c-5p (liver, heart, kidney, spleen, stomach, skeletal muscle, blood, BALF), miR-30d (lung, skeletal muscle), and miR-30e (lung, liver, kidney, urinary bladder, skeletal muscle, blood); members of the miR-181 family, including miR-181b (lung, urinary bladder, stomach, BALF), miR-181c-3p (lung, stomach), miR-181c-5p (urinary bladder, skeletal muscle), and miR-181d (lung, stomach, urinary bladder); and miR-185-3p (kidney, stomach, spleen, skeletal muscle).

By comparing the data obtained in celecoxib-treated mice, either unexposed or exposed to MCS, it is noteworthy that 10 miRNAs (miR-106a, miR-290, miR-381, miR-471, miR-615, miR-758, miR-1247, miR-3070, miR-3085, and miR-3109) were upregulated by celecoxib in the lung of both sham-exposed and MCS-exposed newborn Swiss H mice, while no miRNA was modulated by celecoxib in the lung of both sham-exposed and MCS-exposed adult ICR (CD-1) mice. Although we cannot rule out possible interstrain differences, these results show a sharp difference in the epigenetic response to this NSAID when administered for 6 weeks either to post-weanling mice exposed to MCS since birth or to adult mice in which treatment with celecoxib started shortly before the first exposure to MCS. Such an age-related difference was also confirmed by the finding that 6 miRNAs (miR21/21a, miR-185, miR-335, miR-361, miR-695, and miR-1964) were upregulated by celecoxib in the blood serum of MCS-free Swiss H mice, while all of them were downregulated in the blood serum of young adult ICR (CD-1) mice. Four miRNAs (miR-181-b/c, miR-471, miR-511/511b, and miR-1247) were upregulated by celecoxib in the lung of both MCS-free newborn Swiss H mice and young adult ICR (CD-1) mice. Two miRNAs (miR-185, and miR-335) were downregulated by celecoxib in the blood serum of both sham-exposed and MCS-exposed ICR (CD-1) mice. The same two miRNAs were downregulated by celecoxib in both lung and blood serum of MCS-exposed ICR (CD-1) mice. It is noteworthy that, in MCS-exposed mice, miR-185 was modulated both in lung and blood serum at both low and high celecoxib doses. Therefore, this miRNA represents a blood biomarker that reflects the protective effect exerted by this COX-2 inhibitor in the lung.

There are concerns regarding the cardiotoxicity of several agents used both in cancer treatment and in cancer prevention [46], among which the case of celecoxib is paradigmatic [9, 10]. In addition to the previously discussed formation of bulky DNA adducts in the heart of mice treated with celecoxib, it was of interest to evaluate modulation by this drug of those miRNAs that are known to target the heart. Of the miRNAs analyzed in the present study, miR-308 was upregulated by celecoxib in the blood serum of MCS-free neonatal Swiss H mice, and miR-208 was upregulated in both lung and blood serum of the same mice. In adult ICR (CD-1) mice, miR-30c-5p was downregulated in several organs, among which heart, and in the blood serum of MCS-free mice. Within the same miRNA family, miR-30b was downregulated in the heart of both MCS-free and MCS-exposed mice.

Of the above mentioned miRNAs, miR-30 plays a role in myocardial matrix remodeling [47] and contributes to endoplasmic reticulum stress in cardiac muscle and vascular smooth muscle cells [48]. miR-208 has been included in the list of muscle-specific miRNAs, called myomiRs, and has extensively been investigated both in cardiomyocyte development [49] and heart injury [50]. As inferred from studies in rats, this miRNA has been proposed as a plasmatic biomarker of myocardial injury [51] and other cardiovascular diseases [52]. In addition, miR-208 has been suggested to be a potent therapeutic target for the modulation of cardiac function and remodeling during heart disease progression [53]. From a mechanistic point of view, it has been proposed that downregulation of miR-208 contributes to reactive oxygen species (ROS) production in mouse cardiomyocytes and enhances apoptosis of these cells mainly by targeting p21 [54]. The same considerations may hold true for miR-328, the other miRNA that we found to be increased in the blood serum of celecoxib-treated Swiss H mice. In fact, studies in humans showed that circulating miR-328 could be a potential indicator for heart injury, and the levels of this miRNA are associated with increased risk of mortality or development of acute myocardial infarction [55], cardiac hypertrophy [56], and atrial fibrillation [57].

Our finding that both miR-208 and miR-328 are upregulated in mice treated with celecoxib early in life is new and may contribute to understand the cardiovascular effects produced by this selective COX-2 inhibitor. Accordingly, it may be assumed that maintenance of these miRNAs at an appropriate level in the blood of celecoxib-treated individuals may be taken as a safety indicator in order to avoid possible adverse effects of this drug on the cardiovascular system.

In conclusion, the results of the three studies reported in the present article show that celecoxib is able to attenuate the DNA alterations produced by MCS in mouse lung in terms of bulky DNA adducts and oxidative DNA damage. Moreover, celecoxib counteracted the MCS-related dysregulation of several miRNAs in a variety of organs and body fluids thereby suggesting that this NSAID has pleiomorphic properties. Such a conclusion is in agreement with the hypothesis that NSAIDs may reduce the “inflammogenesis of cancer” not only by interfering with the arachidonic acid cascade but also through multiple mechanisms [2, 58, 59]. CS induces inflammation also by blocking miRNA maturation at the DICER level, thus causing accumulation of miRNA precursors in cytoplasm that trigger TLR (toll-like receptor) activation [60, 61]. The findings of the present studies evaluating genomic and epigenetic biomarkers are consistent with the results of a parallel medium-term bioassay in Swiss H mice in which celecoxib attenuated the MCS-induced alterations of inflammatory nature, the yield of lung adenomas, and progression to cancer in the lung [18].

On the other hand, administration of celecoxib to non-smoking mice resulted in evident molecular alterations. These included the selective formation of bulky DNA adducts in both lung and heart and the dysregulation of a number of miRNAs in various organs, also including those miRNAs that are heart-specific and have been involved in cardiovascular diseases, which may bear relevance in the pathogenesis of the cardiovascular adverse effects of this drug.

## MATERIALS AND METHODS

### General design of the studies

In all 3 studies the mice were divided into the following 4 groups: (*a*) mice kept in filtered air (sham); (*b*) mice treated with celecoxib; (*c*) mice exposed to MCS; and (*d*) mice exposed to MCS and treated with celecoxib. *Study 1* evaluated the levels of bulky DNA adducts and the intensity of oxidative DNA damage in lung, the frequency of micronucleated normochromatic erythrocytes (MN NCE) in the blood, and the expression of miRNAs in both lung and blood of Swiss H mice as related to exposure to MCS for 10 weeks, starting at birth, and administration of celecoxib after weanling (about 4 weeks) until the 10th week of life. Ten mice per group (5 males and 5 females) were used. *Study 2* evaluated the levels of bulky DNA adducts in the lung, liver, and heart of Swiss ICR (CD-1) mice as related to exposure to MCS for 10 weeks, starting at birth, and administration of celecoxib after weanling (about 4 weeks) until the 10th week of life. Twenty mice per group (10 males and 10 females) were used. *Study 3* evaluated the expression of miRNAs in 10 organs, including lung, liver, heart, kidney, spleen, urinary bladder, skeletal muscle (left gastrocnemius), colon, stomach, and brain, and 3 body fluids, including blood serum, bronchoalveolar lavage fluid (BALF), and urines, of two-month old Swiss ICR (CD-1) mice, as related to exposure to MCS for 8 weeks and administration of celecoxib for the same period, starting 3 days before exposure to MCS. Each group was composed of 10 female mice.

### Mice

Strain H neonatal mice, used in *Study 1*, were born at the Animal Laboratory of the National Center of Oncology (Sofia, Bulgaria). The mice were housed in Makrolon™ cages on sawdust bedding and maintained on standard rodent chow (Kostinbrod, Bulgaria) and allowed drinking water ad libitum. Swiss ICR (CD-1) mice, either neonatal (*Study 2*) or two- month old (*Study 3*), were purchased from Harlan Laboratories (San Pietro al Natisone, Udine, Italy). The mice were housed in Makrolon™ cages on sawdust bedding and maintained on standard rodent chow (Teklad 9607, Harlan Laboratories) and tap water *ad libitum*. The animal room temperature was 23 ± 2°C, with a relative humidity of 55% and a 12 h day-night cycle. Housing and treatments of mice were in accordance with NIH, European (2010/63 UE Directive), and institutional guidelines. The issuance of the NIH Office of Laboratory Animal Welfare (OLAW) with the University of Genoa bears the identification number A5899-01 and is effective until February 28, 2021. The Institutional Animal Care and Use Committee (IACUC) protocol was approved by the Fox Chase Cancer Center Committee on April 13, 2015.

### Exposure to MCS

A whole-body exposure of the mice to MCS was achieved as previously described [20, 22], by using 3R4F Kentucky reference cigarettes (College of Agriculture, The Reference Cigarette Program, University of Kentucky, Lexington, KY), having a declared content of 9.4 mg tar, 0.73 mg nicotine, and delivering 12 mg of CO each when burned. MCS was delivered to the exposure chambers by drawing 15 consecutive puffs, each of 60 ml and lasting 6 s. Each daily session involved 6 consecutive exposures, lasting 10 min each, with 1 min intervals during which a total air change was made. Exposure started either within 12 h after birth (*Study 1* and *Study 2*) or at the age of 2 months (*Study 3*) and continued daily for 8-10 weeks. The average total particulate matter (TPM) concentrations in the exposure chambers were 689 mg/m^3^ in *Study 1*, 737 mg/m^3^ in *Study 2*, and 784 mg/m^3^ in *Study 3*.

### Administration of celecoxib

Celecoxib was supplied by the US National Cancer Institute (NCI) via MRIGlobal (Kansas City, MO). Based on a preliminary subchronic toxicity study in Swiss H mice [18], it was decided to incorporate celecoxib in the mouse diet at the dose of 1,600 g/Kg diet, which corresponded to the 80% of the maximum dose that did not produce any bodyloss or sufferance or alterations in the behavior of smoke-free mice after 6 weeks of treatment. Moreover, in *Study 3* two additional groups of female mice, either smoke-free or MCS-exposed, received low dose celecoxib (20 mg/kg diet).

In *Study 1* and *Study 2*, feeding of the drug-containing diet started after weanling (about 4 weeks) and continued until the 10th week of life. In *Study 3*, administration of celecoxib with the diet started 3 days before the first exposure to MCS and continued until the end of the experiment (8 weeks).

### Euthanasia of mice and collection of organs and body fluids

At the end of each experiment, the mice were euthanized. Each mouse was transferred into a separate chamber and isolated from the other mice. The 2013 American Veterinary Medical Association (AVMA) guidelines on euthanasia were followed using slow introduction of CO_2_ asphyxiation, and death was confirmed by absence of respiration and/or heartbeat.

The urine was collected for 8 h and pooled from the mice belonging to each experimental group in *Study 3* by using metabolic cages during the day preceding euthanasia of mice. Immediately after sacrifice, a bronchoalveolar lavage (BAL) was performed and used for preparing BALF, and the blood was collected and used for preparing serum. BALF and blood serum were pooled within each experimental group. The organs reported in the general design of the studies were collected, as previously described [22]. Immediately after collection, each organ or fluid was immersed in RNAlater and kept at -80°C for miRNA analysis. Portions of the organs used in *Study 1* and *Study 2* were stored at -80°C for evaluating DNA damage. Moreover, in order to evaluate the frequency of MN NCE, the blood was collected from the lateral tail vein of 40 neonatal Swiss H mice (20 males and 20 females), used in parallel experiments [18], which were exposed to MCS and/or treated with celecoxib until the 4th month of life.

### Extraction and ^32^P postlabeling analysis of lung DNA

DNA was extracted from tissue specimens (50 mg) collected individually from the lungs of 40 Swiss H mice used in *Study 1* and from lungs, heart and liver of 80 ICR (CD-1) mice used in *Study 2*, as previously described [23]. Bulky DNA adducts, after butanol enrichment, and 8-hydroxy-2’-deoxyguanosine (8-oxo-dGuo) were measured by ^32^P postlabeling as previously described [62].

### Modulation of the systemic MCS genotoxicity

At 4 months of age, immediately after discontinuing exposure to MCS, peripheral blood was collected from the tail lateral vein and smeared onto slides (2 slides/mouse) from 20 mice (10 males and 10 females) per each one of the 4 experimental groups. After staining with May-Grünwald-Giemsa, the frequency of MN NCE was scored by analyzing microscopically 50,000 NCE/mouse.

### Extraction of RNA and analysis of miRNA expression in mouse organs and body fluids

RNA was extracted from mouse organs (miRNeasy, Qiagen, Hilden, Germany) and body fluids (Exiqon's miRCURY™ RNA Isolation Kit – Biofluids (Exiqon, Vedbaek, Denmark) according to the manufacturers’ instructions. The quality of the RNA extracted was evaluated by spectrophotometric analyses using a fiber optic spectrophotometer (Nanodrop ND-1000).

For evaluating the expression of pulmonary miRNAs we used the 7th generation miRCURY LNA™ microRNA Array (Exiqon), which contains 3,100 capture probes covering human, mouse and rat miRNAs. In particular, this array covers 1,135 mouse miRNAs, i.e. the 88.6% of the 1,281 mouse miRNAs listed in miRBase 19. Microarray analyses were carried out as previously described [22].

Microarray data have been recorded in the GEO database (GEO accession No. requested).

### Statistical analysis

Treatment-related differences in body weights, in the frequency of MN NCE, and in the levels of pulmonary DNA adducts and 8-oxo-dGuo, all of them expressed as means ± SE within each group of mice, were evaluated for statistical significance by ANOVA followed by Student's *t* test for unpaired data. miRNA microarray data, after local background subtraction, log transformation, and normalization were analyzed by GeneSpring software (Agilent, Santa Clara, CA), and expression data were median centered by using the GeneSpring normalization option. The statistical significance of the differences between experimental groups was evaluated by means of the GeneSpring ANOVA applied by using Bonferroni multiple testing correction. As inferred from volcano-plot analysis, differences between sets of data were taken as significant when they were both statistically significant (*P* < 0.05) and showed at least a two-fold variation. The overall variability of microarray data, as related to treatments, was evaluated by scatter-plot analysis (SPA), hierarchical cluster analysis (HCA), and principal component analysis (PCA).
